# Modulating autism spectrum disorder pathophysiology using a trace amine-focused approach: targeting the gut

**DOI:** 10.1186/s10020-025-01232-3

**Published:** 2025-05-20

**Authors:** L. Pretorius, J. A. Coetzee, A. P. dos Santos, C. Smith

**Affiliations:** Experimental Medicine Group, Dept Medicine, Stellenbosch University, Parow, South Africa

**Keywords:** β-PEA, Tyramine, Tryptamine, Agmatine, Polyamine, TAAR, Behaviour, Gut-brain, Gut health

## Abstract

Autism spectrum disorder (ASD) affects approximately 1% of the population directly, but also a much higher proportion (family and caregivers) indirectly. Although ASD is characterized by high prevalence of anxiety and poor gastrointestinal health, current treatment strategies are mainly focused on neurological symptomatic treatment, with little to no attention to gut health. Furthermore, many psychiatric drugs used for management of secondary neurological symptoms, are known to exacerbate gut health issues and neurological dysregulation across the gut-brain axis.

Trace amines are neurotransmitter-like substances synthesized endogenously in the human brain – in trace amounts – but also in high abundance by the microbiome. Emerging evidence suggests dysregulation of the trace amine system in ASD. Since trace aminergic signalling is central to regulatory system homeostasis, we hypothesize targeting this system in the ASD context. Given the various sources of trace amines, we suggest that normalization of functional dysbiosis in terms of trace aminergic signalling – rather than microbial compositional dysbiosis – should be a focus in medicines development. In addition, a holistic consideration including also other factors at play in determining trace aminergic signalling outcome – such as receptor binding, enzymatic role players, etc. – is required to fully elucidate and therapeutically modify the pathophysiology of regulatory systems implicated in ASD.

This review firstly provides a brief overview of trace amine dysregulation in ASD for context. Secondly, we formulate our hypothesis on how this may therapeutically address symptomology, with consideration of cellular and molecular mechanism interplay across the gut-brain axis. Finally, we provide a critical assessment of advances in therapeutics development and drug re-purposing, gaps in knowledge and priorities for medicines development going forward.

## Introduction

Despite the fact that non-neurological pathophysiological concerns such as poor gastrointestinal (GI) health are reported in the majority of individuals with autism spectrum disorder (ASD) (Wang et al. 2022a), these are not currently prioritised in the management of ASD. Current treatment strategies are limited to a combination of behavioural, educational, and pharmaceutical therapies for the treatment of neurological symptoms and neurological co-morbidities (anxiety, depression, ADHD). However, commonly prescribed pharmaceuticals, such as risperidone and aripiprazole, may further compromise gut health (Aishworiya et al. 2022), possibly due to their effect to alter gut microbiota composition (Bretler et al. 2019) thereby inducing or exacerbating gut dysbiosis (Angoa-Pérez and Kuhn 2021; Rukavishnikov et al. 2023; Seeman 2021) – although heterogenous study designs have limited conclusions on exact mechanisms at play (Dias et al. 2024). Nevertheless, altered microbial composition and microbially derived metabolite actions in the gut may in turn exacerbate neurological symptoms via gut-brain signalling, suggesting that ASD-associated gut-brain axis alternations can contribute to an ASD phenotype. Indeed, a functional gut-brain axis architecture has been correlated to ASD phenotypic heterogeneity and is characterized by altered macronutrient metabolism that correlates to restrictive dietary patterns, pro-inflammatory cytokine profile and changes in brain-derived gene expression profiles (Morton et al. 2023). This fact emphasises that consideration of gut health should be prioritised in ASD. In addition, these studies (Angoa-Pérez and Kuhn 2021; Rukavishnikov et al. 2023; Seeman 2021) acknowledge that a relative gut dysbiosis may affect the pharmacokinetic profile and efficacy of these drugs.

Acknowledging the heterogeneity of ASD aetiology and symptomology, it would be naïve to propose that a single biochemical pathway may be responsible for the full spectrum of ASD disease pathology. However, we have identified the trace aminergic system, which functions at the level of both the brain and gut (albeit at different levels of abundance), as potential target for modulation in the context of ASD. For example, an altered trace amine load (due to altered microbial composition or consumption of trace amine rich foods) and downstream trace aminergic signalling is anticipated in the ASD context but remains to be confirmed. Importantly, trace aminergic signalling comprises many role players (e.g. trace amines, their receptors and metabolic enzymes) and seems to contribute an additional level of regulation to multiple major regulatory process in the body, such as redox status, inflammatory pathways and neuronal function (Gwilt et al. 2020). While modulation of trace amine signalling is unlikely to modify the genetic foundation of ASD pathology, it may significantly impact psychological and peripheral symptomology, ultimately enhancing overall quality of life and functionality of individuals with ASD.

The purpose of this hypothesis paper is to explore the avenues by which modulation of trace amine signalling may have therapeutic benefit in the ASD context. We first provide a brief summary of known ASD pathologies (neurological, gastrointestinal and mitochondrial). We then provide an integrated discussion of the available literature informing on the potential involvement of aberrant trace amine signalling in the context of ASD symptomology. Finally, we formulate our hypothesis on potential targets for therapeutic intervention in this context and make recommendations for research priorities going forward.

## ASD pathology

### Neurological pathology in ASD

As summarised in Table 1, abnormalities in neuronal connectivity and synaptogenesis may to a large extent account for the primary pathophysiology of ASD. However, the relative contribution of neurotransmitter system abnormalities to primary disease outcome remains to be confirmed. Nevertheless, the most consistent changes related to ASD-associated behaviours seem to be associated with alterations in serotonergic and dopaminergic signalling, but links to other neurotransmitters also exist. For example, decreased gamma-aminobutyric acid (GABA) levels align with the high incidence of anxiety in ASD (Lever and Geurts 2016; Simonoff et al. 2022; Wang et al. 2018a), as well as incidence of seizure (Frye et al. 2016; Tallarico et al. 2023) and mitochondrial functional abnormalities (Frye et al. 2019) in ASD. Importantly, limited available data seems to suggest that central (brain) and peripheral (e.g. circulation, gut) neurotransmitter profiles may differ e.g. evidence for low central vs. high peripheral 5-HT levels (Muller et al. 2016). This suggests another level of (or differential) regulation of neuronal signaling in the peripheral vs. central compartments, but insufficient information limits firm conclusions at this point.

**Table 1:** Neurological pathophysiology of autism spectrum disorder as elucidated in humans (post-mortem analyses and clinical studies) and pre-clinical studies

Clinical evidence		Pre-clinical evidence	
**Neuroanatomical pathologies**			
↑ Brain weight (children)	(Courchesne et al. 2011a)		
Brain overgrowth at earliest ages, but ↓ brain volume at older ages	(Courchesne et al. 2011b)		
↓ Parietal lobe volume at all ages	(Courchesne et al. 1993)		
↑ PFC neuronal number (children)	(Courchesne et al. 2011a)		
↑ Cell-packing density & ↓ neuronal cell size in the limbic system (Hipp, Amg & EC) at all ages	(Bauman and Kemper 1985; Bauman 1991; Raymond et al. 1996)		
↓ Purkinje cell number (neo-cerebellar cortex) at all ages	(Bauman and Kemper 1985; Bauman 1991)	↓ Purkinje cell number (rat motor cortex, NST & Cereb)	(Al Sagheer et al. 2018; Haida et al. 2019)
↑ Dendritic spine densities (cortical pyramidal cells)	(Hutsler and Zhang 2010)		
**Central neurological pathologies**			
Normal developmental ↑ in early childhood 5-HT synthesis capacity is disrupted in ASD	(Chugani et al. 1999)	↓ L-trp, 5-HTP, 5-HT, 5-HIAA levels (rat brain)	(Kong et al. 2021)
		↓ 5-HT levels (rat mid-brain) ↑ 5-HT levels (rat Cereb & pons)	(Ali and Elgoly 2013; Kumar and Sharma 2016)
↑ Serotonergic innervation (Amg, Pir, STC & PHC)	(Azmitia et al. 2011; Lew et al. 2020)	↑ TPH-ir neurite number (rat Rn)	(Wang et al. 2013)
↓ 5-HT_1A/2A_R-binding density - limbic-cortical network (ACC, PCC & FG)	(Brandenburg and Blatt 2019; Oblak et al. 2013)		
↓ SERT density (ACC & FG deep layers)	(Brandenburg and Blatt 2019; Oblak et al. 2013)		
↓ SERT binding capacity	(Makkonen et al. 2008) (Nakamura et al. 2010)	↑ SERT binding (rat Amg)	(Wang et al. 2013)
		↓ Striatal (rat) TH expression	(Cezar et al. 2018)
		↓ TH1-ir neurons (larval ZF diencephalon)	(Baronio et al. 2018)
Striatal DA synthesis unaffected	(Schalbroeck et al. 2021)	↓ DA levels (rat Hipp & mid-brain) ↑ DA (rat FC, Cereb & pons)	(Ali and Elgoly 2013)
		↓ DA levels (adult ZF brain)	(Ricarte et al. 2024)
↓ FDOPA (PFC)	(Ernst et al. 1997)		
↑ D_D2_R mRNA (MSNs in CN & Pu)	(Brandenburg et al. 2020)	↑ D_D2_R expression (rat MSNs in NAc) in adolescence & adulthood ↑ D_D1_R expression (rat MSNs in NAc & Hipp) in adulthood only	(Schiavi et al. 2019)
↑ Frequency of the D_D2_R rs1800498TT genotype	(Hettinger et al. 2012)		
↑ DAT binding capacity	(Nakamura et al. 2010; Xiao-Mian et al. 2005)		
DAT binding capacity unaffected	(Makkonen et al. 2008)		
↑ Excitatory synapse number (upper cortical layers) ↓ Inhibitory synapse number (all cortical layers)	(Vakilzadeh et al. 2024)	↑ Excitation/inhibition ratio (rat DRn)	(Wang et al. 2018b; Zieminska et al. 2018)
GABAergic signal disruption	(Robertson et al. 2016)		
↓ GABA levels (frontal lobe)	(Harada et al. 2011)	↓ GABA levels & ↑ Glu levels (rat Cereb & PFC) ↓ GABA levels (adult ZF brain)	(Kong et al. 2021) (Ricarte et al. 2024)
		↑ GABA & Glu levels (rat FC & Hipp)	(Ali and Elgoly 2013; Zieminska et al. 2018)
↓ GABA_A1/A2/A3/B3_R expression (parietal cortex, PCC & FG)	(Fatemi et al. 2009; Oblak et al. 2011)		
		MAO-A/B knockout mice showed ASD-like phenotype	(Bortolato et al. 2013; Singh et al. 2013)
		↓ Histaminergic neurons & histamine (larval ZF posterior hypothalamus)	(Baronio et al. 2018)
**Psychiatric co-morbidities - anxiety & depression**			
↑ risk for anxiety & depression	(Buck et al. 2014; Gotham et al. 2015; Lever and Geurts 2016)	↑ Anxiety-like behaviours (rats & ZF)	(Ali and Elgoly 2013; Baronio et al. 2018; Han et al. 2022; Kong et al. 2021; Ricarte et al. 2024; Wang et al. 2013, 2018b; Zieminska et al. 2018)
Anxiety-like & depressive-symptom levels in females ↑ at a faster rate throughout adolescence, while males have ↑ levels of depressive symptoms in school age that are maintained into young adulthood.	(Gotham et al. 2015; Uljarević et al. 2020)		
Individuals with intellectual disabilities are less likely to experience anxiety or depression.	(Buck et al. 2014)		
Older adults less often met criteria for social phobia than younger adults.	(Lever and Geurts 2016)	↓ social interaction (rats & ZF)	(Ali and Elgoly 2013; Baronio et al. 2018; Han et al. 2022; Kong et al. 2021; Ricarte et al. 2024; Schiavi et al. 2019; Wang et al. 2013)
**Peripheral neurotransmitter-related pathologies**			
↑ 5-HT (plasma, serum & platelet) & 5-HIAA (serum) levels, associated with severity of ASD	(Aaron et al. 2019; Abdulamir et al. 2018; Hranilovic et al. 2007; Marler et al. 2016; Wang et al. 2020)	↓ L-trp, 5-HTP & 5-HT levels (ZF serum)	(Kong et al. 2021)
↓ PPP 5-HT levels	(Alabdali et al. 2014; Spivak et al. 2004)		
		↓ ZF colonic L-trp, 5-HTP, 5-HT & 5-HIAA levels ↓ 5-HT (rat ileum)	(Kong et al. 2021; Kumar and Sharma 2016)
		↓ ZF faecal L-trp & 5-HTP levels	(Kong et al. 2021)
↑ serum SERT levels	(Aaron et al. 2019; Abdulamir et al. 2018; Marler et al. 2016)		
↓ plasma DA & HVA levels	(Alabdali et al. 2014) (Wang et al. 2020)	↓ DA levels (ZF serum)	(Kong et al. 2021)
SNPs in D_D1_R and D_D2_R as potential risk factors for ASD	(Mariggiò et al. 2021)		
↑ plasma GABA levels	(Alabdali et al. 2014)	↓ GABA & ↑ Glu levels (ZF serum)	(Kong et al. 2021)
VNTR functional polymorphisms in MAO-A gene associated with severity of anxiety in boys with ASD	(Roohi et al. 2009)		

Abbreviations: PFC, prefrontal cortex; Hipp, hippocampus; Amg, amygdala; EC, entorhinal cortex; NST, nigrostriatal pathway; Cereb, cerebellum; 5-HT, serotonin; ASD, autism spectrum disorder; Pir, piriform; STC, superior temporal cortex; PHC, parahippocampal cortex; 5-HT_1/2A_R, serotonin 1/2A receptor(s); ACC, anterior cingulate cortex; PCC, posterior cingulate cortex; FG, fusiform gyrus; SERT, serotonin transporter; DA, dopamine; FDOPA, fluorine-18-labelled fluorodopa; D_D2/D1_R, dopamine D2/D1 receptor(s); MSNs, medium spiny neurons; CN, caudate nucleus; Pu, putamen; DAT, dopamine transporter; GABA, gamma aminobutyric acid; GABA_A1/A2/A3/B3_R, GABA A1/A2/A3/B3 receptor(s); L-trp, L-tryptophan; 5-HTP, 5-hydroxytryptopha; 5-HIAA, 5-hydroxyindoleacetic acid; TPH-ir, tryptophan hydroxylase immunoreactive; TH, tyrosine hydroxylase; TH1-ir, tyrosine hydroxylase 1 immunoreactive; ZF, zebrafish; FC, frontal cortex; NAc, nucleus accumbens; DRn, dorsal raphe nucleus; Glu, glutamate, MAO-A/B, monoamine oxidase-A/B; PPP, platelet poor plasma; HVA, homovanillic acid; SNPs, single nucleotide polymorphisms; VNTR, variable number tandem repeat

### Mitochondrial dysfunction as central mechanism

There is increasing evidence suggesting that neurodevelopmental disorders are affected by mitochondrial dysfunction and the resulting increased oxidative stress load (Anitha et al. 2023; Wang et al. 2022b). Abnormal mitochondrial biomarkers have been reported in 30–50% of individuals with ASD, while the reported prevalence of electron transport chain (ETC) abnormalities in immune cells are as high as 80% (Frye et al. 2019; Giulivi et al. 2010; Napoli et al. 2014; Rossignol and Frye 2012). Importantly, these characteristic mitochondrial functional abnormalities have been demonstrated in human ASD studies (summarised in Table 2) and reflect a generally reduced mitochondrial respiration and functional capacity. Unfortunately, a relative paucity of data exists on mitochondrial function in ASD specifically. This is a significant oversight, since not all data generated in the brain vs. the periphery align (Table 2). As such, a more holistic profile of mitochondrial dysfunction across compartments may significantly contribute to our understanding of ASD as mitochondrial disease, as well as identification of therapeutic targets.

**Table 2:** Functional abnormalities reported in mitochondria in the autism spectrum disorder context

Pathophysiology			References
**Mitochondrial respiratory chain**			
	Centrally	Peripherally	
Complex I	↓	↓ / ↑	(Anitha et al. 2013; Bu et al. 2017; Chauhan et al. 2011; Elesawy et al. 2022; Gu et al. 2013b; Tang et al. 2013)
Complex II	↓	↓	
Complex III	↓	↓	
Complex IV	↓	↓ / ↑	
Complex V	↓	↓	
↓ Mitochondrial membrane potential			(Elesawy et al. 2022)
↓ ATP production			
**Mitochondrial dynamics**			
Fission > Fusion ↓ expression of mitochondrial fusion proteins (OPA1, MFN1 and MFN2) ↑ expression of mitochondrial fission proteins (Fis1, Drp1)			(Anitha et al. 2013; Tang et al. 2013)
Mitophagy ↓ hypothesized (but evidence relatively lacking)			(Tang et al. 2013)
Trafficking and localization ↓ accessory protein genes (MTX2 and NEFL). Information sparse, inconclusive			(Anitha et al. 2013)
**Redox**			
Increased reactive oxygen species centrally and peripherally			(Bu et al. 2017; Trinchese et al. 2022; Wang et al. 2024a, b)
Altered glutathione redox system ↓ GSH, ↑ GSSG, ↓GSH/GSSG			(Elesawy et al. 2022; Gu et al. 2013a; James et al. 2004; Melnyk et al. 2012; Trinchese et al. 2022)
↓ primary antioxidant enzyme activity (GPx, SOD2 (mitochondrial), CAT)			(Altun et al. 2018; Elesawy et al. 2022; Tang et al. 2013; Wang et al. 2024a, b)
↑ lipid peroxidation (MDA), markers of oxidative damage to DNA and proteins, advanced glycation end-products.			(Chauhan et al. 2004; Elesawy et al. 2022; Tang et al. 2013)

Abbreviations: ATP, adenosine triphosphate; OPA1, optic atrophy 1; MFN1/2, mitofusin, 1/2; Fis1, mitochondrial fission 1 protein; Drp1, dynamin-related protein 1; MTX2, metaxin 2; NEFL, neurofilament protein; GSH, glutathione; GSSG, glutathione disulfide; GPx, glutathione peroxidase; SOD, superoxide dismutase; CAT, catalase; MDA, malondialdehyde

Furthermore, the contribution of mitochondrial dysfunction to poor GI health in ASD is well-recognised (Rose et al. 2018a). Mitochondria were suggested to be the biological link between environmental stressors (toxicants, medications, microbial secretory products) and enterocyte dysfunction (Frye et al. 2015). In this case – in addition to genetically determined mitochondrial abnormalities in ASD (Frye et al. 2024) – the high environmental challenge endured by the gut enterocytes specifically, could further exacerbate mitochondrial dysfunction, rendering the enterocytes even less functional (Basivireddya et al. 2002; Guerbette et al. 2022; Madsen et al. 1995; Moschandrea et al. 2024). While evidence is not yet conclusive, increased intestinal permeability in patients with ASD has been suggested by several groups (de Magistris et al. 2010; Esnafoglu et al. 2017; Teskey et al. 2021). This would result in a higher antigenic load and increased immune activation, contributing to low-grade GI and systemic inflammation. Clearly, mitochondrial function (and its normalisation) should be an important consideration in ASD management.

### ASD-associated gastrointestinal dysfunction

**Fig. 1:**
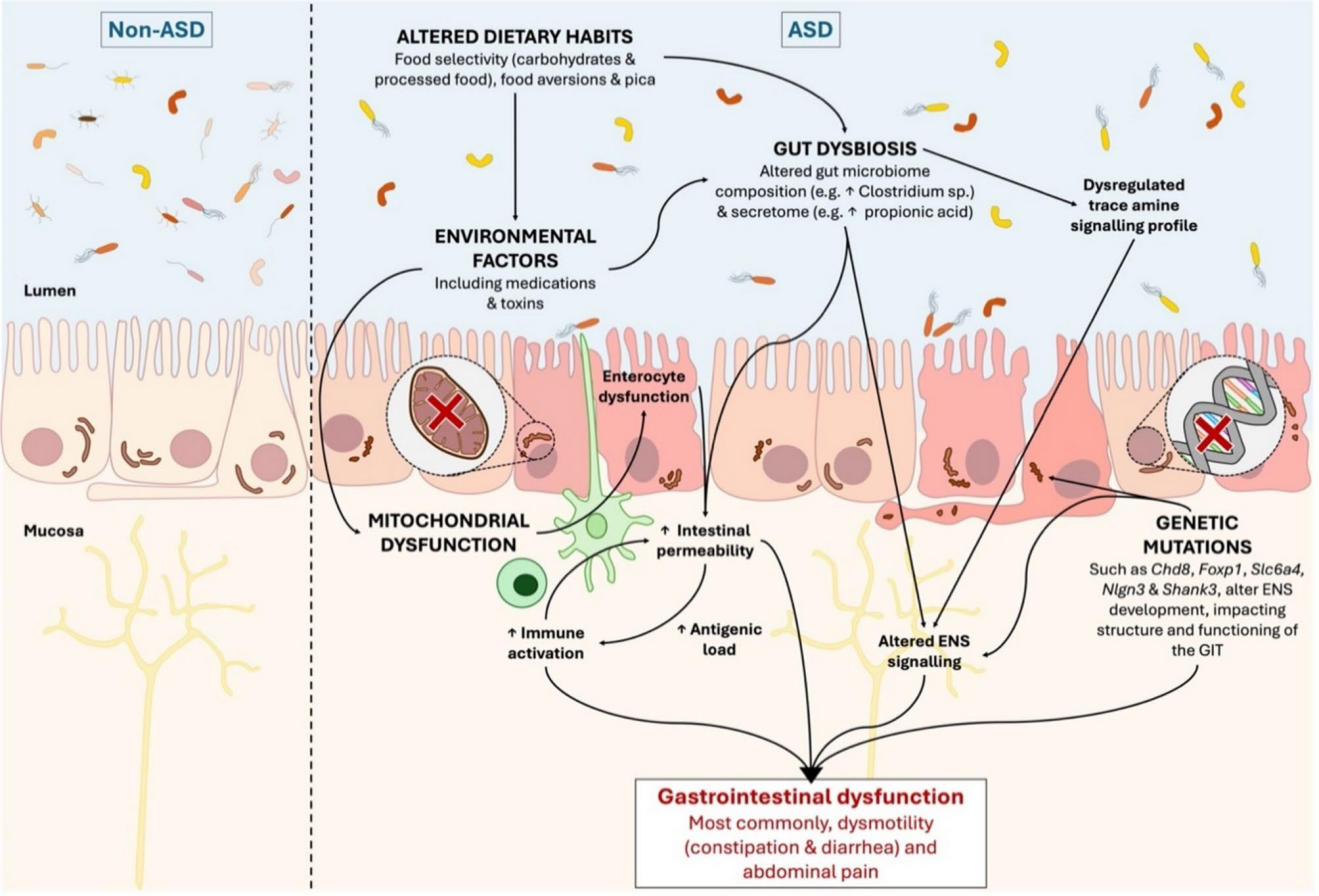
Potential pathogenesis of gastrointestinal dysfunction in autism spectrum disorder (ASD) as extrapolated from non-ASD literature. Abbreviations: ENS, enteric nervous system; GIT, gastrointestinal tract; MET, mesenchymal-epithelial transition factor. Red ‘X’ indicates genetic abnormalities that result in altered structure and/or function

We present a summary of mechanisms likely to be involved in ASD-associated GI dysfunction in Fig. 1. As illustrated, ASD-associated poor gut health is the net outcome of interplay of multiple contributors. For example, central nervous system (CNS)-relevant gene mutations have been proposed to affect the development of the enteric nervous system (ENS) in ASD (Bernier et al. 2014; Campbell et al. 2009; Fröhlich et al. 2019; Hosie et al. 2019; James et al. 2019; Leembruggen et al. 2020; Margolis et al. 2016), impacting its structure and function throughout life (Niesler and Rappold 2021). Of particular interest, ASD-associated serotonin transporter (SERT) polymorphisms are likely to disrupt serotonin (5-HT) metabolism and alter the regulation of mucosal immune responses (Shajib et al. 2017), as well as the activation of enteric reflexes that underlie gut motility, secretion, and sensation (Mawe and Hoffman 2013). However, not all individuals with these mutations exhibit GI dysfunction, aligning with the notion of genetic and environmental interplay in aetiology of poor gut health in ASD.

Moving on to diet, individuals with ASD present with significant food aversions and selectivity that typically lead them to consume a diet high in carbohydrates and processed foods (Cermak et al. 2010; Esposito et al. 2023). An unbalanced diet exacerbate GI symptomology (Valenzuela-Zamora et al. 2022) primarily via disruption of gut microbiome homeostasis (Singh et al. 2017; Zhang 2022), resulting in altered gut microbial composition and secretome profiles (Coretti et al. 2018; De Angelis et al. 2013; Finegold et al. 2002; Hughes et al. 2018; Iglesias-Vazquez et al. 2020; Wang et al. 2012). As previously mentioned, the balance of microbial secretory products and their metabolites, may significantly impact mitochondrial function, and thus GI function. Furthermore, many of these microbial secretory products – specifically trace amines – are also produced endogenously in the brain, where they play important roles in neurological function. We believe that going forward, a gut-brain approach – and with that, trace amine signalling in particular – should be a major focus in the design of therapeutic strategies in ASD.

## Interconnectedness of brain and gut in ASD: a role for trace amines

While the benefit of several therapeutic strategies (prebiotic/probiotic therapy, faecal microbial transplant and dietary interventions) aimed at regulating gut microbial composition remains to be conclusively proven (Fattorusso et al. 2019; Geier et al. 2011; Grimaldi et al. 2017; Grossi et al. 2016; Kang et al. 2013; Ooi et al. 2015), we propose a somewhat different approach to gut-brain modulation in ASD, shifting the focus from *compositional* to *functional* dysbiosis. In line with this, metabolomic investigations of serum, faeces and urine have revealed altered abundance of a number of molecules in ASD that are of microbial origin (Needham et al. 2021; Sharon et al. 2019). Furthermore, many individuals with ASD present with metabolic imbalances, immune dysregulation, GI dysfunction and altered gut-brain signalling, all of which are significantly impacted by gut microbial metabolites – these include both non-trace amine (e.g. short chain fatty acids (SCFA) originating from bacterial fermentation of dietary carbohydrates, (Rogers et al. 2016)) and trace aminergic role players (e.g. ρ-tyramine, β-phenethylamine and tryptamine) (Gwilt et al. 2020). This emphasises the potential impact of gut microbial secretory products (and thus a functional dysbiosis) on ASD pathophysiological outcome.

Since trace amines are involved in the regulation of several pertinent contributors to comorbid ASD pathophysiology, altered trace aminergic signalling – and the therapeutic normalisation thereof – will be discussed in gut-vs-brain and redox/mitochondrial contexts below.

### Trace aminergic signalling pathways of relevance to ASD

Currently, due to a relative paucity of information, firm conclusions in terms of potential trace amine-based causality in primary ASD pathology is not possible, and thus this is not the topic of this review. Rather, the focus will be on the potential to therapeutically target trace amine signalling for improvement of symptomology of ASD across the gut-brain axis.

#### Modulation of neurotransmitter signalling pathways

**Fig. 2:**
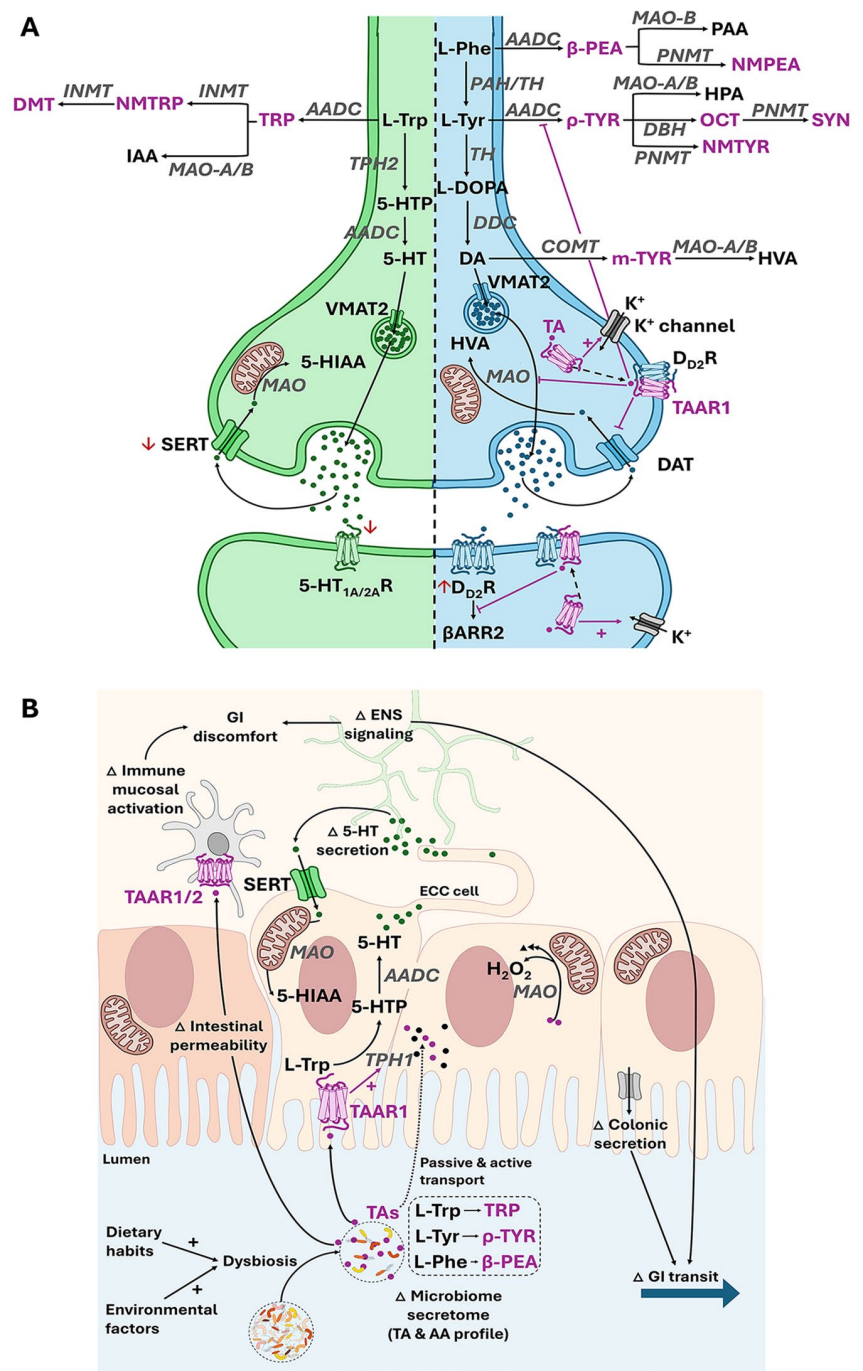
Proposed trace aminergic regulation in the brain (**A**) and gut (**B**), as it relates to ASD-related pathology and symptomology. Information in frame A reflects data from human studies only, but due to the paucity of human data on the gut, information in frame B was also summarised from preclinical studies. The trace aminergic system is represented in purple, with known ASD pathologies indicated with the use of red arrows. All enzymes are in grey. Abbreviations: ENS, enteric nervous system; 5-HT, serotonin; TAAR, trace amine-associated receptor; AADC, aromatic L-amino acid decarboxylase; MAO-A/B, monoamine oxidase-A/B; 5-HTP, 5-hydroxytryptophan; TPH1/2, tryptophan hydroxylase 1/2; L-Trp, L-tryptophan; L-Tyr, L-tyrosine; L-Phe, L-phenylalanine; TRP, tryptamine; r-TYR, r-tyramine; β-PEA, β-phenethylamine; GI, gastrointestinal; TA, trace amine; AA, amino acid; ASD, autism spectrum disorder; DMT, N,N-Dimethyltryptamine; NMTRP, N-methyltryptamine; IAA, Indoleacetic acid; INMT, indolethylamine-N-methyltransferase; SERT, serotonin transporter; 5-HT_1A/2A_R, serotonin 1 A/2A receptor; 5-HIAA, 5-hydroxyindoleacetic acid; PAH, phenylalanine hydroxylase; TH, tyrosine hydroxylase; PAA, phenylacetic acid; NMPEA, N-methylphenethylamine; NMTYR, N-methyltyramine; PNMT, phenylethanolamine N-methyltransferase; OCT, octopamine; SYN, synephrine; HPA, 4-hydroxyphenylacetaldehyde; DBH, dopamine beta-hydroxylase; m-TYR/3-MT, 3-Methoxytyramine; COMT, catechol-O-methyltransferase; DAT, dopamine transporter; HVA, homovanillic acid; L-DOPA, levodopa; DDC, DOPA decarboxylase; DA, dopamine; VMAT2, vesicular monoamine transporter 2; D_D2_R, dopamine receptor D2; βARR2, beta-arrestin-2; ECC, enterochromaffin

Given the interconnectedness of neurotransmitter and trace amine metabolic pathways, altered trace amine metabolism may significantly affect neurotransmitter flux. This is illustrated in Fig. 2, where substantial overlap in the regulation of neurotransmitter signalling by the trace aminergic system exists in both brain and gut compartments, suggesting a potential for therapeutic intervention at both sites via trace aminergic modulation. For example, metabolomics of ASD rodent liver samples (as proxy for general metabolic status) reported increased intensity of β-phenethylamine (β-PEA) (Cao et al. 2023), which – given the common precursor (L-phenylalanine) for β-PEA and dopamine (DA) – may suggest altered substrate availability for DA synthesis and subsequent activity, although correlations between liver and central levels and the implications for dopaminergic activity in ASD remains to be confirmed. In addition, DA synthesis is also dependent on ρ-tyramine metabolism, from their shared precursor, L-tyrosine. Although dysregulated ρ-tyramine levels have not been reported in ASD, a slight increase in faecal m-tyramine (a catabolite downstream from DA) was reported for ASD vs. typically developed controls (Needham et al. 2021), suggesting altered dopaminergic metabolism. Also, ρ-tyramine metabolites octopamine and synephrine have been linked to the ASD hallmarks aggression (Jia et al. 2021; Sherer et al. 2020) and adverse redox outcome (Ribeiro et al. 2019), respectively. This suggests potential alterations in ρ-tyramine flux towards production of octopamine and synephrine rather than DA (see Fig. 2) may indicate another avenue for dysregulation at the cost of DA synthesis in ASD. Furthermore, trace amine associated receptor (TAAR)-1 agonism (functions of both β-PEA and ρ-tyramine) is known to reduce presynaptic DA signalling via TAAR1/dopamine-D2 receptor (D_D2_R) dimerization-induced inhibition of presynaptic DA release and reuptake via dopamine transporter (DAT) (Halff et al. 2023). Given that ASD is characterised by reduced DA signalling, increased TAAR1 activation – and subsequent TAAR1/D_D2_R dimerization – may therefore play a role in altered signalling cascades in ASD. Moreover, TAAR1 is expressed in nearly all immune and neuroimmune cells (Fleischer et al. 2018) and its activation and/or dimerization with TAAR2 has been linked to peripheral pro-inflammatory outcome (Babusyte et al. 2013; Panas et al. 2012), accumulation of intracellular calcium and adverse redox outcome (Moiseenko et al. 2024). Thus, skewing of phenylalanine and tyrosine metabolism to favour β-PEA and ρ-tyramine signalling, may contribute significantly to the neurological, oxidative and pro-inflammatory characteristics of ASD.

The intricate nature of dysregulation at this level, was further illustrated in a rodent valproic acid (VPA)-model for ASD, which reported the “dopaminergic hypofunction” effect to be TAAR1- and sex-specific, with females reportedly less affected, which is aligned with the higher male prevalence of ASD reported clinically (Hara et al. 2015). In line with this, oestradiol was recently reported to attenuate ρ-tyramine-associated GI dysregulation in the context of intestinal inflammation (Pretorius and Smith 2023). While it remained to be confirmed how these direct actions of exogenous oestradiol (to reduce ρ-tyramine-associated inflammation and oxidative stress and improve tight junction protein expression) in a gut context may connect to reduced female vs. male prevalence of ASD, a lower probability of females to develop intestinal permeability has been postulated as an etiological mechanism that could be attributed to the protective effect of oestrogen in the ASD context (El-Ansary et al. 2020). In addition to this, the effect of oestradiol to affect the sex dimorphism of ASD through sex-dependent microbially-derived metabolite production has also been suggested (Hao et al. 2020). These data may suggest that females may be protected from ASD outcomes associated with ρ-tyramine or its metabolites, although this remains to be confirmed. Importantly, the interplay of hormones and trace amine signalling warrants further investigation - especially given the relative lack of inclusion of females in mechanistic studies until relatively recently – and as recently implied (Hokanson et al. 2024), this may indicate that ASD in males and females may require different treatment approaches to address different therapeutic targets, and as such considering the impact of sex in ASD treatment is imperative.

Furthermore, although decreased 5-HT signalling has long been established in ASD, very little progress has been made in terms of therapeutics development (Pourhamzeh et al. 2022), highlighting the complexity of dysregulation in this system. Trace amines may also contribute to ASD symptomology here. For example, pineal dysfunction has been implicated in ASD; apart from accounting for the reduced melatonin levels reported in ASD, abnormal pineal metabolism of N, N-dimethyltryptamine (DMT) - a breakdown product of the trace amine tryptamine and thought to act via both TAARs and serotonin 2 A receptor (5-HT_2A_R) - may be responsible for DMT accumulation, which in turn results in abnormal neurogenesis, including the aberrant synaptic organisation characteristic of ASD (Shomrat and Nesher 2019). This suggests potential alterations in L-tryptophan flux towards production of tryptamine and DMT rather the 5-HT in ASD (see Fig. 2) that may at least in part, underpin some of the central serotonergic abnormalities associated with ASD, although this remains to be confirmed. However, this outcome contrasts the anxiolytic benefit ascribed to low level DMT in human studies (Jacob and Presti 2005) and again highlights the requirement for intricate regulation of absolute levels of any of the trace amines, to avoid adverse outcome.

Moreover, altered trace amine levels have been reported in the context of common neurological ASD comorbidities. Of interest, seemingly contradictory roles have been reported for β-PEA. In addition to its undesired link to ASD symptomology already mentioned, both ADHD and depression are associated with decreased urinary β-PEA and its major metabolite phenylacetic acid (Baker et al. 1991; Sabelli and Javaid 1995; Zametkin et al. 1984), while β-PEA supplementation ameliorated corticosterone-induced depression in a murine model (Lee et al. 2020). These data suggest that modulatory effects of β-PEA are dose- or receptor-dependent. Given the complexity of ASD in terms of severity spectrum and heterogeneity of comorbid symptomology, causality if unlikely to rest with one dysregulated trace amine. Unfortunately, limited parameter panel and sampling diversity used for trace amine quantification, limits interpretation on dysregulation of the balance between different role players at pathway or tissue compartment levels, or how these limited observations in one sample type (e.g. urine or faeces) may relate to pathology elsewhere (e.g. brain). Thus, it remains unclear whether or not trace amines are accurate biomarkers of ASD pathology or comorbid symptomology. Nevertheless, these studies support the notion that neurotransmitter signalling may be normalised via modulation of trace amine signalling.

#### Modulation of gut function

Moving on to the gut context, the notion that trace amines are physiologically active in the gut is strongly supported by faecal metabolomic studies reporting trace amines at relatively high (micromolar) levels (Jacobs et al. 2016; Kisuse et al. 2018; Ponnusamy et al. 2011; Santoru et al. 2017; Zhai et al. 2023), as well as by data from several in vivo and ex vivo studies demonstrating altered gut function (e.g. increased gut transit via promotion of colonic ion and 5-HT secretion) after supplementation with either trace amines at physiologically relevant concentrations, or specific trace amine-producing bacteria (Bhattarai et al. 2018; Williams et al. 2014; Yano et al. 2015; Zhai et al. 2023). Since modulation of intestinal secretion and motility can have profound effects on factors that affect intestinal homeostasis (luminal pH, mucosal immune response, and delivery of nutrients to gut microbes and host enterocytes), it is not surprising that many GI disorders (with high prevalences of comorbid neuropsychiatric symptomologies) report altered faecal trace amine levels. For example, in various irritable bowel syndrome (IBS) cohorts, levels of β-PEA have been reported as increased (Zhai et al. 2023) or decreased (Han et al. 2022), while faecal tryptamine levels seem consistently increased (Ponnusamy et al. 2011; Zhai et al. 2023). Moreover, in patients with inflammatory bowel disease (IBD), increased levels of β-PEA, putrescine and cadaverine have been reported in a Crohn’s disease sub-group (Jacobs et al. 2016; Santoru et al. 2017), while increased levels of cadaverine, trimethylamine-N-oxide and ρ-tyramine were reported in the ulcerative colitis sub-group (Santoru et al. 2017). It is important to note that changes in trace amine levels have not only been associated with changes in specific bacterial groups, but also disease severity and even co-morbid symptomology. Indeed, in patients with diarrhoea-predominant IBS, elevated levels of faecal β-PEA and tryptamine were associated with severity of diarrhoeal symptoms, while elevated peripheral 5-HT levels (induced via β-PEA/tryptamine-TAAR1 signalling) directly implicates altered trace aminergic signalling in IBS pathology (Zhai et al. 2023). In addition, tryptamine levels were reportedly elevated in an IBS group with co-morbid depression (approximately 30% of the IBS cohort) when compared to healthy controls (Han et al. 2022), with tryptamine/tryptophan ratio relatively increased with increased depression severity in IBS patients, suggesting that co-morbid neurological disorders may be exacerbated by specific alterations in trace amine load in the gut. Of significance in the medicines development context, a rescue effect (via experimental TAAR-1 inhibition) was demonstrated in pseudo germ-free mice colonized with diarrhoea-predominant IBS (IBS-D)-derived microbiota (Zhai et al. 2023), suggesting that normalisation of trace aminergic signalling (and thus also neurotransmitter signalling) can occur *despite* microbial compositional dysbiosis. While these specific pathways have not yet been investigated in ASD, elevated *blood* 5-HT was the first biomarker identified in autism research (reviewed by (Gabriele et al. 2014), suggesting that a related pathology may exist to increase peripheral 5-HT and cause GI symptomology. Moreover, given the important role of 5-HT in neuronal formation and presynaptic balance (keeping in mind that ASD is associated with neuronal malformations and dysregulated neurotransmission), the contribution of altered ENS signalling or metabolite load from gut-associated changes cannot be underplayed in the ASD context.

In the context of immune system activation (and thus inflammatory and redox outcome), altered trace amine homeostasis has already been hypothesised to result in hyperactivity of the immune system, specifically mucosal immunity (Christian and Berry 2018) – which is known to be dysregulated in ASD when co-morbid GI symptomologies are also present (Ashwood et al. 2004; Ashwood and Wakefield 2006; Butera et al. 2024; Rose et al. 2018a). Again, these studies suggest that trace amine homeostasis is crucial for GI homeostasis. However, even in ASD individuals without clinical GI symptoms, low level dysregulation of cellular processes such as mitochondrial function would still be present in the gut, which may benefit from trace amine modulation.

#### Targeting mitochondria

**Fig. 3:**
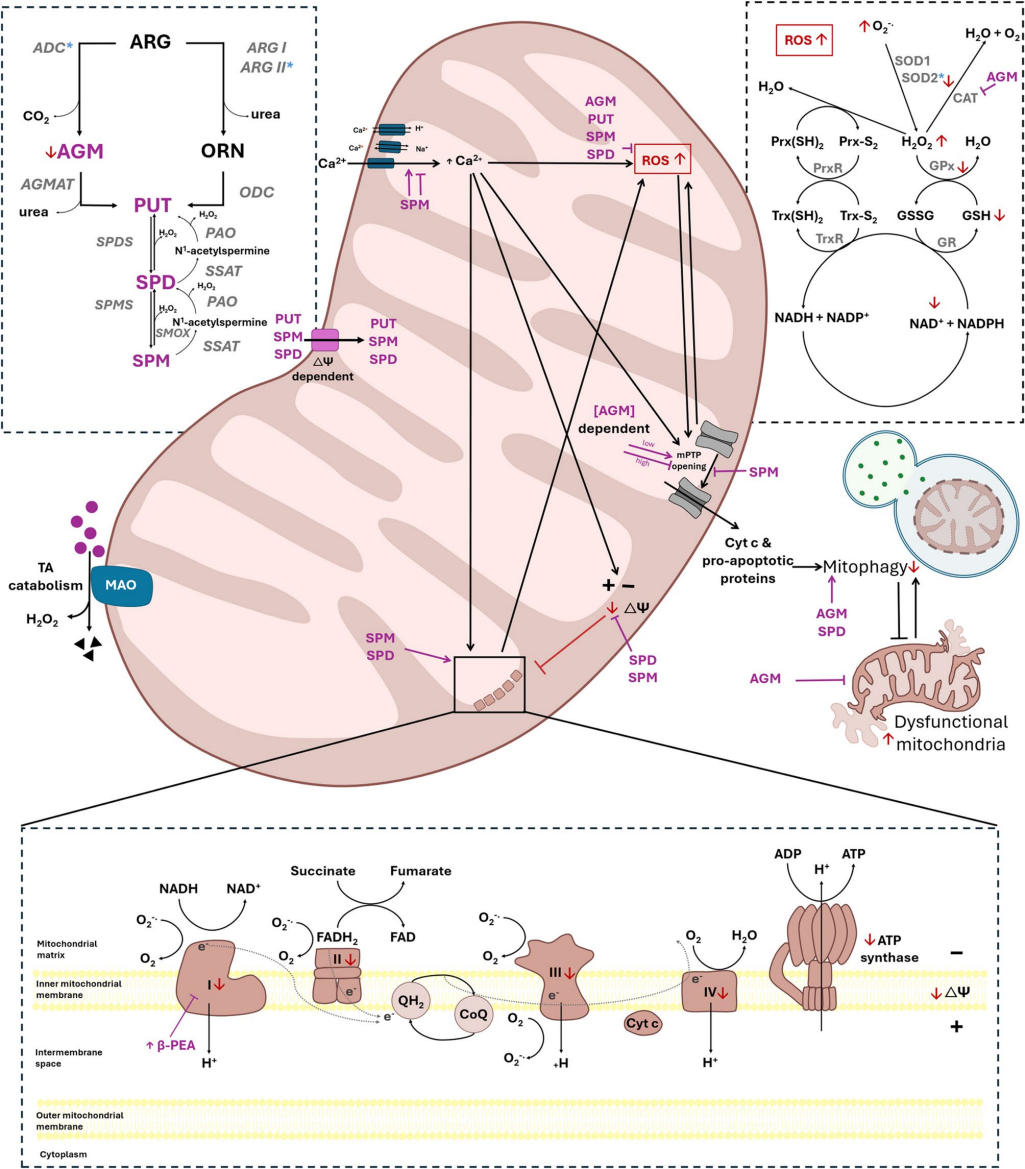
Trace aminergic regulation in the mitochondria, as it relates to ASD-related pathology and symptomology. Mitochondrial location of enzymes is indicted with a blue asterisk (*). Red font indicates abnormalities associated with ASD. Abbreviations: ARG, L-arginine; ARG I / II, arginase I / II; ORN, ornithine; PUT, putrescine; AGM, agmatine; ODC, ornithine decarboxylase; PAO, polyamine oxidase; SSAT, spermidine/spermine-N(1)-acetyltransferase; SPDS, spermidine synthase; SPMS, spermine synthase; SMOX, spermine oxidase; AGMAT, agmatinase; SPM, spermine; SPD, spermidine; TA, trace amines; MOA, monoamine oxidase; ROS, reactive oxygen species; SOD 1 / 2, superoxide dismutase 1 / 2; PrxR, peroxiredoxin reductase; Prx(SH)_2_, reduced peroxiredoxin; Prx-S_2_, oxidized peroxiredoxin; CAT, catalase; GSSG, glutathione disulfide; GSH, glutathione; GR, glutathione reductase; NAD^+^, oxidized Nicotinamide adenine dinucleotide; NADPH, nicotinamide adenine dinucleotide phosphate; Trx-S_2_, oxidized thioredoxin; Trx(SH)_2_, reduced thioredoxin; TrxR, thioredoxin reductase; mPTP, mitochondrial permeability transition pore; I – V, mitochondrial electron transport chain complex I – V; Cyt, c – cytochrome c; ATP, adenosine triphosphate; ADP, ddenosine diphosphate; NADH, reduced nicotinamide-adenine dinucleotide; FADH_2_, reduced flavin adenine dinucleotide; FAD, flavin adenine dinucleotide; β-PEA, β-phenylethylamine; QH_2_, dihydroxyquinone; CoQ, coenzyme Q; GPx, glutathione peroxidase; GR, glutathione reductase; ∆Ψ, mitochondrial membrane potential

In the context of another common comorbid ASD symptom – anxiety – the role of trace amines in determining redox outcome is clearly illustrated. For example, the role of agmatine (plasma levels of which are decreased in ASD (Esnafoglu and Irende 2018) and its metabolites in the regulation of mitochondrial function, is illustrated in Fig. 3. In the context of ASD specifically, murine gestational agmatine treatment was shown to normalise autistic behaviour – including anxiety – in offspring (Chen et al. 2024), which was associated with increased dephosphorylation of ERK and CREB in the ERK/CREB/BDNF pathway. In a non-autism model, agmatine was shown to reduce anxiety via its inhibition of nitric oxide synthase (Gawali et al. 2017), suggesting a redox-associated mechanism. In line with this, in a rodent VPA model of autism, both prenatal and postnatal acute agmatine administration were recently reported to rescue impaired social behaviour in male offspring (Khoram-Abadi et al. 2024; Kim et al. 2017). While no benefit was observed in terms of plasma total antioxidant capacity, reduced malonaldehyde activity were reported in this model, pointing toward some redox benefit. In line with this, agmatine administration in ASD has been shown to influence cellular energy metabolism to decrease glycolysis in favour of oxidative mechanisms, increasing mitochondrial activity (Hipkiss 2018).

As discussed for β-PEA, the requirement for fine control of absolute levels of trace amines – and their metabolites – is also evident from the agmatine literature. For example, in a model of putrescine depletion, lower spermidine/spermine and glutamate/GABA ratios were also strongly associated with anxiety-like behaviour (Gupta et al. 2009). In the same study, higher spermidine levels were associated with good water maze performances and decreased anxiety, whereas spermine had the opposite effect, suggesting differential roles for spermidine and spermine in behavioural outcome. In contrast, an increased spermidine/spermine ratio (resulting from a spermine synthase gene mutation in mice) was linked to increased anxiety, downregulated mitochondrial oxidative phosphorylation and ribosomal protein synthesis in the cerebral cortex, as well as impaired mitochondrial bioenergetics in isolated primary fibroblasts (Akinyele et al. 2024), emphasizing the differential dose-dependent effect of and importance of regulation of these polyamines on behavioural and functional outcome. While these studies suggest that conversion of agmatine into its downstream metabolites may be the key to beneficial outcome, more research is required to understand homeostatic thresholds that result in differential outcomes.

Similarly, in the context of intestinal inflammation, while agmatine administration in human intestinal cell cultures – where normal clearance of agmatine via conversion into its metabolites were likely suboptimal – had detrimental effects on endothelial cell health and barrier integrity (Pretorius and Smith 2022), the same treatment showed protective benefit in an in vivo model – where pathways to metabolise agmatine were intact (Pretorius and Smith 2023). Together, these reports highlight the importance of considering not only the levels of trace amines themselves in development of therapeutics, but also the levels and activity of the enzymes involved in their synthesis or metabolism, as well as the abundance of their downstream metabolites. To our knowledge, potential dysregulation of trace amine-relevant enzymes has not been studied in the context of ASD.

Regarding cellular redox status, it is well known that polyamines contribute to cellular oxidative balance (Lenis et al. 2017). On the one hand, spermine and spermidine reportedly mediate protection against oxidative damage (Belle et al. 2004; Rider et al. 2007), while on the other hand elevated levels of polyamines seem to have toxic effects and are associated with several diseases (Agostinelli 2020; Fiori and Turecki 2008). The basis of this toxicity is likely oxidative stress/damage caused by the production of reactive species (such as hydrogen peroxide and acrolein) during polyamine catabolism. Similarly, the effects of agmatine on cellular redox status and mitochondrial functionality seem to be dependent on a homeostatic balance and regulation of its pleiotropic effects. For example, agmatine has been related to neuroprotective outcomes (Kotagale et al. 2019) via reduction in oxidative stress and preservation of mitochondrial integrity and function (Condello et al. 2011; El-Sayed et al. 2019). However, a hormetic effect of agmatine on the mitochondrial permeability transition mechanism has been reported in rat liver samples (Martinis et al. 2020), with mitochondrial permeability transition induced by low concentrations but inhibited by high concentrations. This suggests a very complex role of agmatine on mitochondrial physiology but emphasises the need for homeostatic regulation to achieve cellular redox homeostasis.

A further consideration is that some trace amines are metabolised by monoamine oxidase (MAO) enzymes, a catabolism process that also produces hydrogen peroxide as byproduct (Bortolato et al. 2008). In healthy cells, this oxidizing agent is neutralised by catalase or glutathione peroxidase into non-toxic H_2_O. However, any abnormality in antioxidant capacity or activity (such as in diseased states), could lead to oxidative stress-induced cellular damage. Since MAO is mitochondrial bound, mitochondria may be subject to excessive reactive oxygen species if increased trace amine metabolic flux were to occur (i.e. in states of increase trace amine load). This, in conjunction with the fact that MAO inhibition seems to prevent mitochondrial dysfunction (Sorato et al. 2014; Venegas et al. 2024), suggests that altered trace amine metabolism may contribute to, or exacerbate, mitochondrial dysfunction. In addition, *dose-dependent* effects of β-PEA to generate hydroxyl radicals in vitro and in vivo via significant inhibition of NADH-ubiquinone oxidoreductase activity have been reported (Sengupta and Mohanakumar 2010. This suggests that β-PEA-induced inhibition of oxidative phosphorylation may result in excess hydroxyl radical generation (Sengupta and Mohanakumar 2010) and strengthens the point that altered trace amine load and subsequent metabolism may contribute to mitochondrial dysfunction.

## Trace amine-focused therapeutics development: do we know enough?

From the reviewed literature, it is clear that dysregulated trace amine signalling underpins ASD pathology along multiple pathways. A major limitation to trace amine-focused medicines development in ASD, is the relative absence of GI data on trace amines. For conditions with a clear gut-brain link – such as ASD – execution of more studies including parallel assessment at gut and brain level should be a priority. Methodological advancements allowing the assessment of concentrations of comprehensive panels of trace amines across different matrices, are only now emerging (de Bruyn et al. 2024; Henning et al. 2024) and will contribute substantially to our understanding of dysregulation in this regard, in ASD. Furthermore, ASD research data including females are minimal and scattered – inclusion of sex as consideration in research going forward will not only advance management of female individuals on the spectrum but may also provide clues for therapeutics development.

Currently, two viable therapeutic approaches seem to have been identified, namely TAAR1 agonism and agmatine administration. Firstly, in terms of agmatine, we have already discussed its potential. However, also in this context, many gaps in our knowledge still remain. For example, while direct NMDA receptor antagonism of agmatine has been established as therapeutic mechanism centrally (Khoram-Abadi et al. 2024; Kim et al. 2017), the role of agmatine in the gut itself or from the gut (i.e. affecting change indirectly at locations outside of the gut) may be more complex. For example, oral administration of agmatine in a model of experimental depression, was shown to confer benefit via normalisation of gut barrier integrity (cellular junction proteins), but also neuroinflammation and serotonergic signalling in the hippocampus and prefrontal cortex (Rahangdale et al. 2024). A potential limitation to this study was that depression was induced via total eradication of the gut microbiome with antibiotics, so that these benefits of agmatine is not yet conclusive in a more physiologically relevant scenario. Thorough simultaneous investigation of mechanisms and outcomes in an ASD-specific model – both centrally and peripherally – is still lacking.

Secondly, TAAR1 agonism (using the experimental agonist RO5263397) has been linked to beneficial effects on neurological symptomology – such as frustration, irritability and aggression – in a rodent VPA model (L. Wang et al. 2024a, b). In a non-ASD context, TAAR1 antagonism by methamphetamine was shown to decrease cellular calcium flux (Proulx et al. 2024), which supports TAAR1 agonism in ASD to potentially improve mitochondrial respiration. In addition, other experimental compounds have been reported to show benefits in terms of metabolic regulation (specifically glycaemic control and satiety) (Dedic et al. 2024; Raab et al. 2016) that could reduce risk for cardiovascular disease, which is increased in ASD. However, while TAAR1 agonism has also been shown to delay stomach emptying – which at first glance may be the mechanism for satiety (Dedic et al. 2024; Milanovic et al. 2024) – this effect may in fact pose risk in ASD, where repetitive behaviour in terms of dietary patterns may result in relative ineffectiveness of satiety to reduce food intake. Indeed, the same study (Milanovic et al. 2024) reported a significant increase in gut disorders, and we have previously shown that delayed stomach emptying without a change in dietary habits (in rodents) resulted in significant morbid cardiovascular complications (Smith and Krygsman 2014). On a technical note, the majority of preclinical protocols producing beneficial results for TAAR1 agonism, employed intraperitoneal delivery as mode of administration. This bypasses the gut and potential gut-based effects of TAAR1 agonism, e.g. its previously demonstrated effects on serotonin signalling (Zhai et al. 2023). Thus, as is the case for agmatine, given the narrow focus of most preclinical investigations, a holistic picture of benefit vs. risk across tissues and compartments is still lacking. Furthermore, reports of contradictory inflammatory outcome after experimental modulation (by both agonists and antagonists) of TAAR1 signalling (Barnes et al. 2021; Gwilt et al. 2019; Polini et al. 2023), suggests that several contributing factors need to be considered in development of drugs therapeutically targeting TAAR1. These include specificity of ligand, tissue, cellular localisation and dose, as well as the nature of TAAR1 dimerisation. Studies of this scope is not available yet, but the hope is that drug trial designs will incorporate investigations at multiple levels to address these significant gaps in our knowledge.

In terms of progress on re-purposing existing drugs, the versatility of the TAAR1 binding pocket highlights its potential to recognise various ligands (Jiang et al. 2024), suggesting that drug re-purposing may be a feasible avenue for the therapeutics discovery in this context. Indeed, drug re-purposing strategies have led to the identification of several TAAR1 agonists, such as asenapine (atypical antipsychotic), fenoldopam (D_1_R partial agonist), guanfacine and guanabenz (both α_2_-adenoreceptor agonists) (Cichero et al. 2023; Shajan et al. 2024). The action of asenapine seems relatively broadly targeting (several neurotransmitter receptors), but other TAAR1 agonists with more specific actions, warrant consideration within the complex ASD scenario, as outcome may be relatively more predictable for these drugs. Nevertheless, a complexity that remains, is the fact that many TAAR1 agonists have lower selectivity for TAAR1 than other monoaminergic G-protein coupled receptors, increasing the risk of off-target effects. As such, besides conventional side effects (most commonly somnolence, headaches and hypotension), off-target receptor activation should be considered. In this case multiple receptors signalling and the related crosstalk should be investigated in the ASD context to confirm drug re-purposing applicability.

In terms of the role(s) of other TAARs in ASD, a study using gene express ion characteristic analyses and weighted gene co-expression network analyses, identified *taar7h* and *taar7b* as hub genes that significantly downregulated neuroactive ligand-receptor interaction pathways in hippocampal samples in VPA-treated rats (Huang et al. 2016), implicating TAARs and related gene regulatory networks as risk factors in ASD. Since *taar7* is a pseudogene in humans, the relevance of these findings is still unclear. However, peptidome analysis revealed increased serum TAAR6 levels in children with ASD vs. controls (Yang et al. 2018), which may represent the translation from the rodent data, but more research is required before firm conclusions can be made.

Finally, an area of substantial neglect is our understanding of the extent to which enzymes involved in trace amine synthesis and metabolism, may be affected in ASD. Isolated enzymes have been investigated in pre-clinical models, e.g. elucidating the decreased levels of hippocampal glutamic acid decarboxylase enzyme (GAD67) as potential role player in the decreased GABA-signalling in VPA-associated ASD in rodents (Win-Shwe et al. 2018), but a holistic profile of potential enzyme dysregulation eludes. An added complexity here is that different isoforms of enzymes exist in different body compartments. For example, in contrast to the mentioned rodent data, *increased* plasma GAD65 levels were previously associated with severe autism in children (Vazifekhah et al. 2022), which complicates integrated interpretation of small data sets across different models. Also here, methodology has been a limiting factor, with population-wide gene-association studies yielding limited success (Buttenschon et al. 2009; Yu et al. 2016). However, in the current era of -omics-based research, significant strides should be possible in terms of elucidating dysregulated enzymatic processes, as well as the roles of environmental factors such as pharmaceuticals.

Together, these reports highlight the importance of a comprehensive assessment including all potential role players, i.e. levels of neurotransmitters and trace amines themselves, but also enzymes important for their synthesis or degradation, as well as the receptors through which they signal, across multiple tissue compartments, for a holistic understanding of the pathology that requires normalisation. Without this, biomarker-driven therapeutics development is not possible. Furthermore, returning to the heterogeneous nature of ASD symptomology, once more comprehensive preclinical data on therapeutic mechanisms has been generated, the next step would be assessment of the general applicability of any particular drug across the spectrum of ASD symptomatic diversity and severity, to determine the need for individualisation of therapeutics.

## Conclusion

Large gaps still exist in the ASD literature, but some common maladaptation in the trace amine niche is clear, highlighting the potential to target this system for therapeutic modulation. Further knowledge gain in terms of trace amine metabolism, as well as receptor and enzymatic role players, may refine drug discovery approaches by elucidating exact mechanisms at play. Nevertheless, preclinical intervention studies can already contribute to therapeutic development by assessing the feasibility of achieving neurological modulation in ASD via intervention at the level of the gut. We propose that this may be achieved via modulation of trace amine signalling within the gut, using “clean” models such as larval zebrafish or germ-free rodents, where the relative contribution of the gut microbiome can be experimentally manipulated and accounted for. Given the known different scales in terms of trace amine abundance in the brain vs. the gut, the use of such models would be particularly useful in assessing “off-target” effects in compartments or organs other than the primary target.

## Data Availability

No datasets were generated or analysed during the current study.

## Abbreviations

5-HIAA: 5-hydroxyindoleacetic acid
5-HT: Serotonin
5-HT_2A/1A_R: Serotonin 1/2A receptor
5-HTP: 5-Hydroxytryptophan
AA: Amino acid
AADC: Aromatic L-amino acid decarboxylase
ACC: Anterior cingulate gyrus
ADC: Agmatine decarboxylase
ADHD: Attention deficit hyperactivity disorder
ADP: Adenosine diphosphate
AGM: Agmatine
AGMAT: Agmatinase
Amg: Amygdala
βARR2: Beta-arrestin-2
ARG: L-Arginine
ARG I/II: Arginase I/II
ASD: Autism spectrum disorder
ATP: Adenosine triphosphate
BDNF: Brain-derived neurotrophic factor
CAT: Catalase
Cereb: Cerebellum
CN: Caudate nucleus
CNS: Central nervous system
COMT: Catechol-O-methyltransferase
CoQ: Coenzyme Q
CREB: cAMP-response element binding protein
Cyt c: Cytochrome c
D_D1/D2_R: Dopamine D1/2 receptor
DA: Dopamine
DAT: Dopamine transporter
DBH: Dopamine beta-hydroxylase
DDC: DOPA decarboxylase
DMT: N, N-Dimethyltryptamine
DRn: Dorsal raphe nucleus
Drp1: Dynamin-related protein 1
EC: Entorhinal cortex
ECC: Enterochromaffin cell
ENS: Enteric nervous system
ERK: Extracellular signal-regulated kinases
ETC: Electron transport chain
FAD/FADH_2_: Flavin adenine dinucleotide
FC: Frontal cortex
FDOPA: Fluorine-18-labelled fluorodopa
FG: Fusiform gyrus
Fis1: Mitochondrial fission 1 protein
GABA: Gamma-aminobutyric acid
GABA_A1/A2/A3/B3_R: GABA A1/A2/A3/B3 receptor(s)
GAD: Glutamic acid decarboxylase enzyme
GI: Gastrointestinal
GIT: Gastrointestinal tract
Glu: Glutamate
GPx: Glutathione peroxidase
GR: Glutathione reductase
GSH: Glutathione
GSSG: Glutathione disulfide
Hipp: Hippocampus
HPA: 4-Hydroxyphenylacetaldehyde
HVA: Homovanillic acid
I – V: Mitochondrial electron transport chain complex I - V
IAA: Indoleacetic acid
IBD: Inflammatory bowel disease
IBS-D: Diarrhoea predominant IBS
IBS: Irritable bowel syndrome
INMT: Indolethylamine-N-methyltransferase
L-DOPA: Levodopa
L-Phe: L-Phenylalanine
L-Trp: L-Tryptophan
L-Tyr: L-Tyrosine
m-TYR/3-MT: 3-Methoxytyramine
MAO-A/B: Monoamine oxidase-A/B
MDA: Malondialdehyde
MET: Mesenchymal-epithelial transition factor
MFN1/2: Mitofusin 1/2
MnSOD: Manganese superoxide dismutase
mPTP: Mitochondrial permeability transition pore
mRNA: Messenger RNA
MSNs: Medium spiny neurons
mtDNA: Mitochondrial DNA
MTX2: Metaxin 2
NAc: Nucleus accumbens
NAD^+^/NADH: Nicotinamide adenine dinucleotide
NADP^+^/NADPH: Nicotinamide adenine dinucleotide phosphate
NEFL: Neurofilament protein
NMPEA: N-Methylphenethylamine
NMTRP: N-Methyltryptamine
NMTYR: N-Methyltyramine
NST: Nigrostriatal pathway
OCT: Octopamine
ODC: Ornithine decarboxylase
OPA1: Optic atrophy 1
ORN: Ornithine
PAA: Phenylacetic acid
PAH: Phenylalanine hydroxylase
PAO: Polyamine oxidase
PCC: Posterior cingulate cortex
β-PEA: β-Phenethylamine
PFC: Prefrontal cortex
PHC: Parahippocampal cortex
Pir: Piriform
PNMT: Phenylethanolamine N-methyltransferase
PPP: Platelet poor plasma
Prx(SH)_2_/Prx-S_2_: Peroxiredoxin reduced/oxidised
PrxR: Peroxiredoxin reductase
Pu: Putamen
PUT: Putrescine
QH_2_: Dihydroxyquinone
ROS: Reactive oxygen species
SCFA: Short chain fatty acid
SERT: Serotonin transporter
SMOX: Spermine oxidase
SNPs: Single nucleotide polymorphisms
SOD1/2: Superoxide dismutase 1/2
SPD: Spermidine
SPDS: Spermidine synthase
SPM: Spermine
SPMS: Spermine synthase
SSAT: Spermidine/spermine N1-acetyltransferase
STC: Superior temporal cortex
SYN: Synephrine
TA: Trace amine
TAAR1/2/6/7: Trace amine associated receptor 1/2/6/7
TH: Tyrosine hydroxylase
TH1-ir: Tyrosine hydroxylase 1 immunoreactive
TPH1/2: Tryptophan hydroxylase 1/2
TPH-ir: Tryptophan hydroxylase immunoreactive
TRP: Tryptamine
Trx(SH)_2_/Trx-S_2_: Thioredoxin reduced/oxidised
TrxR: Thioredoxin reductase
r-TYR: R-Tyramine
VMAT2: Vesicular monoamine transporter 2
VNTR: Variable number tandem repeat
VPA: Valproic acid
ZF: Zebrafish
